# Abdominal radiographs in the emergency department: current status and controversies

**DOI:** 10.1002/jmrs.307

**Published:** 2018-12-02

**Authors:** Ashish Chawla, Wilfred C. G. Peh

**Affiliations:** ^1^ Department of Diagnostic Radiology Khoo Teck Puat Hospital Singapore Singapore

**Keywords:** Abdomen, body, clinical site, general

## Abstract

This editorial is discussing about the indiscriminate use of abdominal radiographs in the emergency department in general, with focus on value of the erect abdominal radiograph for the diagnosis of mechanical bowel obstruction and paralytic ileus.

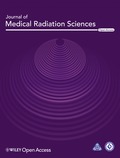

This present issue of Journal of Medical Radiation Sciences features a study by Geng et al assessing the usefulness of the erect abdominal radiograph, in addition to a supine radiograph, in the diagnostic work‐up of adult patients presenting with suspected intestinal obstruction.^1^ Conventional teaching stresses the identification of air‐fluid levels on erect radiographs as a feature of bowel obstruction but this sign has its own limitations. Geng et al. concluded that there is no statistically significant difference in diagnostic accuracy to be gained by adding an extra erect radiograph for identifying mechanical bowel obstruction and/or paralytic ileus in adults presenting with acute abdominal pain. The authors rightly pointed out that performing an erect radiograph may be very inconvenient for elderly and ill patients. Moreover, extra radiographs add to the radiation dose that goes against the ALARA (as low as reasonably achievable) principle. Hence, it is useful to reevaluate the rationale behind this additional radiograph. One limitation of this retrospective study is the focus on only two common indications of abdominal radiograph in the emergency department. In clinical practice, the emergency physicians are frequently unsure of the exact cause of acute abdomen and radiographs may reveal causes of acute abdomen other than intestinal obstruction or paralytic ileus. Nevertheless, Geng et al. have shown that evaluation of these two causes of acute abdomen do not require an erect radiograph.^1^

The diagnostic value of abdominal radiographs is limited but still continues to be recommended as an important component of the diagnostic imaging pathway of acute abdomen. The Royal College of Radiologists (RCR) guidelines about the indications of abdominal radiographs in the emergency department consist of the following: clinical suspicion of obstruction, acute exacerbation of inflammatory bowel disease, palpable mass (specific circumstances), constipation (specific circumstances), acute and chronic pancreatitis (specific circumstances), sharp/poisonous foreign body, smooth and small foreign body, for example, coin, battery (specific circumstances) and blunt or stab abdominal injury (specific circumstances).^2^ Morris‐Stiff et al. concluded that the diagnostic yield of the abdominal radiographs is significantly higher when the RCR guidelines are strictly followed.^3^ In a review of the use of abdominal radiographs in the emergency department, Smith et al. suggested limiting the abdominal radiograph to suspected cases of bowel obstruction and abdominal foreign body.^4^ The American College of Radiology (ACR) has also defined the utility of abdominal series in adult patients with abdominal distension, bowel obstruction, paralytic ileus, foreign bodies, urinary calculi, pneumoperitoneum, post‐placement of the medical device and postoperative patients.^5^ The RCR and ACR guidelines are based on ALARA but are not absolutely clear about the utility of the erect radiographs in the setting of acute abdomen. Our own literature search reveals contradictory results about the usefulness of erect radiographs in bowel obstruction. Field et al. suggested that the erect abdominal radiograph does not provide additional information in the diagnosis of intestinal obstruction or perforation, additional to that obtained from the supine abdominal radiograph and erect chest radiograph.^6^ On the other hand, Lappas et al. demonstrated that the air‐fluid levels seen on erect abdominal radiograph are extremely useful in diagnosis of small bowel obstruction and differentiation of severity of obstruction.^7^

The radiation dose for an abdominal radiograph (0.7 mSv) is high compared to chest radiograph (0.02 mSv). Two abdominal radiographs imply an overall radiation dose of 1.4 mSv. Although widely available, the radiation dose in a standard CT of the abdomen is in range of 10–20 mSv, limiting its use as a screening modality. Apart from the radiation dose, having an additional and possibly unnecessary investigation has financial implications and unwanted workload on the health care system. The low sensitivity and specificity of abdominal radiographs have prompted research in the feasibility of low‐dose CT of the abdomen in the emergency department. Nguyen et al. described the performance of low‐dose CT of the abdomen in comparison to abdominal radiographs.^8^ The results are promising but despite technological advances, the radiation dose remains at 2–3 mSv. The advantage of CT is that it serves a one‐stop imaging modality for most of the causes of the acute abdomen, including rare abnormalities. The continuous advancements in technology will definitely help in further reduction in radiation dose in near future. The advent of artificial intelligence is also expected to dramatically cut down the radiation dose.

Abdominal radiographs should be judiciously used in the emergency department, following established guidelines. We agree with the results of the present study by Geng et al. that an erect abdominal radiograph can be omitted from radiological investigations performed in adult patients suspected to have bowel obstruction or paralytic ileus.^1^ Reviewing the RCR and ACR guidelines for radiography of the acute abdomen in adult patients, it appears that except for those with suspected pneumoperitoneum which will benefit from an additional erect chest radiograph, a supine abdominal radiograph will suffice for all the other indications. This is with the assumption that the radiographs will be interpreted by someone who is well‐trained and competent! We await further data on the utility of developing advanced investigations such as low‐dose CT of the abdomen.

## Conflict of Interest

The authors declare no conflict of interest.

## References

[1] Geng WZ , Fuller M , Osborne B , Thoirs K . The value of the erect abdominal radiograph for the diagnosis of mechanical bowel obstruction and paralytic ileus in adults presenting with acute abdominal pain. J Med Radiat Sci 2018; 65: 259–66. 10.1002/jmrs.299 PMC6275248 30039624

[2] iRefer Guidelines, RCR Version 8.0.1, May 2017 Available from: https://www.irefer.org.uk/guidelines

[3] Morris‐Stiff G , Stiff RE , Morris‐Stiff H . Abdominal radiograph requesting in the setting of acute abdominal pain: Temporal trends and appropriateness of requesting. Ann R Coll Surg Engl 2006; 88: 270–4. 16719997 10.1308/003588406X98586 PMC1963673

[4] Smith JE , Hall EJ . The use of plain abdominal x rays in the emergency department. Emerg Med J 2009; 26: 160–3. 19234001 10.1136/emj.2008.059113

[5] American College of Radiology . ACR‐SPR practice guideline for the performance of abdominal radiography. Available from: http://www.acr.org/-/media/ACR/Files/Practice-Parameters/radabd.pdf?la=en.

[6] Field S , Guy PJ , Upsdell SM , Scourfield AE . The erect abdominal radiograph in the acute abdomen: Should its routine use be abandoned? Br Med J (Clin Res Ed) 1985; 290: 1934–6. 10.1136/bmj.290.6486.1934 PMC1416036 3924315

[7] Lappas JC , Reyes BL , Maglinte DD . Abdominal radiography findings in small‐bowel obstruction: Relevance to triage for additional diagnostic imaging. Am J Roentgenol 2001; 176: 167–74. 11133561 10.2214/ajr.176.1.1760167

[8] Nguyen LK , Wong DD , Fatovich DM , et al. Low‐dose computed tomography versus plain abdominal radiography in the investigation of an acute abdomen. ANZ J Surg 2012; 82: 36–41. 22507493 10.1111/j.1445-2197.2010.05632.x

